# Volatile anesthetics-induced neuroinflammatory and anti-inflammatory responses

**DOI:** 10.1186/2045-9912-3-16

**Published:** 2013-08-01

**Authors:** Franziska E Blum, Zhiyi Zuo

**Affiliations:** 1 Department of Anesthesiology, University of Virginia Health System, 1 Hospital Drive, PO Box 800710, Charlottesville, VA 22908-0710, USA

## Abstract

Volatile anesthetics have been the major anesthetics used clinically for more than 150 years. They provide all components of general anesthesia and are easy to be applied and monitored with modern equipment and technology. In addition to having anesthetic property, volatile anesthetics have multiple other effects. Many studies have clearly shown that volatile anesthetics can reduce systemic and local inflammatory responses induced by various stimuli in humans and animals. On the other hand, recent animal studies have shown that volatile anesthetics may induce mild neuroinflammation. These dual effects on inflammation may have significant biological implications and are briefly reviewed here.

## Introduction

Volatile anesthetics were first introduced into clinical use in 1842 [1]. They have been the mainstay general anesthetics for millions of patients each year. The commonly used volatile anesthetics in current clinical practice include isoflurane, sevoflurane and desflurane [1]. Halothane is no longer used in the U.S.A. but may be used in developing countries [2].

Various theories have been developed over the years to explain the general anesthesia induced by volatile anesthetics. Most researchers now believe that anesthetics bind particular proteins and modify their functions. These effects ultimately result in anesthetic action [1]. A variety of protein targets have been identified for volatile anesthetics. These include ion channels, muscarinic acetylcholine receptors, opioid receptors, α_2_-adrenergic receptors, 5-hydroxytryptophan (HT)_2A_ receptors and N-methyl-D-aspartate (NMDA) receptors [3]. Volatile anesthetics provide all components of general anesthesia including unconsciousness, amnesia, analgesia and muscle relaxation [1]. Apart from the anesthetic property, these anesthetics have found to have multiple biological effects, such as the modulation of inflammation [1,4]. Recently, volatile anesthetics have found to cause mild neuroinflammation [5]. Since inflammation is a fundamental pathological process involved in virtually all diseases acquired after birth, for example, Alzheimer’s and Parkinson’s disease [6,7], these effects of volatile anesthetics on inflammation may have significant biological implications.

### Overview of the inflammatory process

#### Definition of inflammation

Acute inflammation can be defined clinically by the classical signs: pain, heat, redness, swelling and loss of function [8]. On a cellular and molecular level, the inflammatory process is a complex cascade involving inflammatory cells, such as mast cells and neutrophils, and inflammatory factors including cytokines [9].

In contrast to inflammation, infection requires the invasion of a host’s bodily tissues by disease causing organisms, their multiplication, the reaction of host tissues to these organisms and the toxin they produce [8]. Obviously, infection often induces inflammation.

#### Overview of the inflammatory process by using neuroinflammation as an example

When a tissue is injured or infected, a significant amount of pathogenic molecules are produced. These molecules activate dendritic cells, mastocytes, histiocytes and macrophages that reside in the tissues. Dendritic cells are primarily involved in the initiation of the immune response by presenting processed antigens to other cells [10]. Mastocytes, histiocytes and macrophages release inflammatory mediators, such as inflammatory cytokines, to cause the signs of inflammation.

Microglial cells are macrophages residing in the central nervous tissues. They are involved in the neuroinflammatory process. Activation of microglial cells leads to an increase of nitric oxide (NO) via inducible nitric oxide synthase (iNOS) [11]. NO inhibits mitochondrial respiration and induces protein modification, such as S-nitrosylation and nitration [11]. The increased iNOS and NO production can lead to increased production of interleukin (IL)1β (IL-1β), tumor necrosis factor (TNF)-α and interferon-γ (INF-γ) from various cells including microglial cells and neurons [5,12]. TNF-α then triggers activation of caspase 8, which leads to cleavage of caspase 3 to result in cell death [11]. Furthermore, TNF-α can activate NADPH oxidase that produces reactive oxygen species (ROS) from microglial cells, mediates the release of additional proinflammatory factors, and is involved in the induction of caspase-dependent neuronal apoptosis as well as the induction of glutamate release from various brain cells including neurons, astrocytes, dendritic cells and microglia [11,13].

Intracellular signaling molecules that regulate cytokine production and iNOS expression include the mitogen activated protein kinases (MAPK). They are extracellular signal-regulated kinase (ERK 1/2), C-Jun N-terminal kinase (JNK 1/2/3) and p38 kinase (p38) [11]. These kinases lead to the activation of transcription factors, such as nuclear factor κ-light-chain-enhancer of activated B cells (NF-κB), signal activator and transducer of transcription-1 (STAT-1) and activator protein-1 (AP-1), to regulate gene expression [11].

In addition, intracellular calcium is also a significant signaling molecule in the inflammatory cascade [7].

### The anti-inflammatory responses of volatile anesthetics

#### The effects on systemic inflammation

We have shown that exposure of rat NR8283 macrophages to 2% isoflurane for 1 h at 30 min before the application of lipopolysaccharide (LPS) plus INF-γ reduced macrophage injury and NO production. These effects were found to be protein kinas C-dependent [14].

Fuentes et al. found a significant reduction of LPS-induced increase of serum TNF-α, IL-6, and IL-10 in mice exposed to 2% isoflurane for 1 h [15,16]. LPS is an initiator of inflammation [16,17]. This isoflurane effect may be due to the inhibition of NF-κB [15,16]. As a result, mice exposed to isoflurane after a lethal dose (20 mg/kg) survived better than those without isoflurane exposure [16].

Chiang et al. applied 1.4 minimum alveolar concentrations (MAC) isoflurane for 2 h (from 1 h before and 1 h after zymosan application) to mice. Zymosan was used to induce peritonitis. This isoflurane application reduced the number of leukocytes in the peritoneal lavage and the production of inflammatory mediators in the lavage cells [18].

A recent study showed that adult male rats exposed to 2% sevoflurane or 1.5% isoflurane for 30 min before cecal ligation and puncture (CLP) had better survival rates than rats exposed to oxygen only. The CLP procedure was done under ketamine, xylazine and fentanyl anesthesia. The pre-exposure to sevoflurane and isoflurane also reduced the plasma IL-1β, IL-6 and TNF-α levels after CLP. These results suggest that a pre-exposure to volatile anesthetics induces systemic anti-inflammatory effect [19].

#### The anti-inflammatory effect in various organs

Volatile anesthetics have anti-inflammatory effects in various organs, such as the nervous system, the heart, the respiratory system and the kidneys [4,17,20,21].

We have shown that exposure of the C8-B4 mouse microglial cells to various concentrations of isoflurane for 1 h at 30 min before the application of LPS plus INF-γ reduced microglial injury, NO production and glutamate release [22]. In another study, we showed that delayed exposure to 2% isoflurane for up to 2 h after the application of LPS plus INF-γ attenuated C8-B4 mouse microglial injury and NO production. This form of isoflurane treatment also reduced iNOS expression in the mouse brains [23]. These results suggest that isoflurane can inhibit microglial activation. These isoflurane effects on microglial cells may be mediated by activation of protein kinase C [22].

We recently showed that application of 2% isoflurane for 1 h after brain ischemia attenuated the increased NF-κB activation and the production of IL-1β and IL-6 in the ischemic brain tissues. This delayed isoflurane exposure also improved neurological outcome, which may be mediated by the inhibition of IL-1β expression because isoflurane no longer induced neuroprotection in the IL-1β deficient mice [4]. These results provide strong evidence of inhibition of brain ischemia and reperfusion-induced neuroinflammation by isoflurane and its role in neuroprotection.

Hu et al. reported that neutrophils added to rat hearts lost their ability to cause cardiac dysfunction when they were pretreated with 1 MAC sevoflurane or isoflurane for 15 min [20]. Isoflurane and sevoflurane reduced superoxide production from neutrophils by 29% and 33%, respectively. Isoflurane at 0.25 MAC partly inhibited the effects of neutrophils on cardiac functions [20].

Giraud et al. have shown that rats under halothane anesthesia (1% or 1.5% for 4 h) had a 55% reduction of intratracheal LPS-induced recruitment of polymorphonuclear neutrophils (PMN) and a 60% decrease of IL-6, TNF-α and monophage inflammatory protein (MIP-2) in the bronchial lavage and lungs compared to those under thiopental anesthesia [17].

Similar to the findings in other organs, volatile anesthetics have anti-inflammatory effects in the kidneys. Sevoflurane application (2.2% for 4 to 16 h) reduced TNF-α-induced injury of HK-2 cells, a human kidney proximal tubule cell line. Sevoflurane also attenuated the NF-κB activation and inflammatory mediator expression in these cells [21].

#### Human evidence

In addition to animal studies, clinical studies have suggested an anti-inflammatory effect of volatile anesthetics.

Sevoflurane at 2% has been reported to inhibit the function of inflammatory cells by decreasing macrophage-1 antigen receptor expression on granulocytes and PMN in human blood circulating in the extracorporeal circulation circuit. However, sevoflurane had no effect on leukocyte aggregation formation and cytokine release [24].

A study by Nader et al. [25] published in 2004 supports the previously mentioned findings that volatile anesthetics have systemic anti-inflammatory effects shown in animals. The authors had 21 patients anesthetized by propofol-based total intravenous anesthesia. They were randomized into two groups. One group (11 patients) received 2% sevoflurane in the cardioplegia solution (sevoflurane group) and the other group did not (control group). Serum TNF-α was not detectable in the sevoflurane group and the serum IL-6 levels were significantly lower in the sevoflurane group compared to the control group [25]. Similar results were observed by Kawamura et al. [26]. Nader et al. [25] also found that patients in the sevoflurane group had better cardiac function immediately after the cardiopulmonary bypass.

Mahmoud et al. [27] found that alveolar and plasma concentrations of IL-8 and TNF-α were significantly lower in patients undergoing thoracic surgical procedures under 1 MAC isoflurane anesthesia compared to patients who received propofol (4 – 6 mg/kg/h)-based anesthesia [27]. Similarly, De Conno et al. [28] investigated the modulatory effect of sevoflurane on inflammation in 54 patients undergoing thoracic surgery. They found that 1 MAC sevoflurane reduced the levels of TNF-α, IL-6, IL-8 and monocyte chemoattractant protein 1 in the lavage fluids after one lung ventilation compared with patients under propofol anesthesia.

### Neuroinflammation induced by volatile anesthetics

Recently, we showed that exposure to 1.2% isoflurane for 2 h caused a small increase of IL-1β and activated caspase 3 in the hippocampi of both young adult and elderly rats. This isoflurane exposure also leaded to cognitive impairment. Lidocaine, a local anesthetic with anti-inflammatory property, inhibited isoflurane-induced IL-1β increase and cognitive impairment. Also, isoflurane did not induce cognitive impairment in the IL-1β deficient mice. These results suggest that isoflurane induces neuroinflammation that then leads to cognitive impairment [5,29]. Similar to our study, a more recent study showed that exposure of 6-day old mice to 3% sevoflurane for 2 h each day for 3 days increased IL-6 in the brain tissues and resulted in cognitive impairment of these mice when they were more than one month old. These IL-6 increase and cognitive impairment were blocked by ketorolac, an anti-inflammatory reagent [30]. Despite of these lines of evidence in animals, evidence for volatile anesthetics-induced neuroinflammation is lacking up till now.

Little is known for the mechanisms of volatile anesthetics-induced neuroinflammation. Isoflurane has been shown to open the blood brain barrier [31]. This can increase the permeation of intravascular substances to the brain tissues. A recent study showed that exposure of H4 human neuroglioma cells to 2% isoflurane or 4.1% sevoflurane for 6 h activated NF-κB [32]. Since NF-κB is a transcription factor known to increase inflammatory cytokine production [33], activation of NF-κB is presumably a mechanism for volatile anesthetics-induced neuroinflammation.

### Prospective

Many animal studies have shown that volatile anesthetics can inhibit inflammatory process induced by various stimuli including ischemia and inflammation inducing agents. This effect is often translated into organ protection. Similar findings have been obtained in humans. However, limited animal data have suggested that volatile anesthetics can induce neuroinflammation in the absence of other stimuli. This effect is yet to be verified in humans. Nevertheless, current evidence indicates dual effects of volatile anesthetics on inflammatory process: they can inhibit inflammation induced by various potentially harmful stimuli and induce neuroinflammation in the absence of stimuli. Since volatile anesthetics are often used to provide anesthesia for surgery (a stimulus), the anti-inflammatory effect may be what we usually see with volatile anesthetic use in the clinical practice. Understandably, the type and the length of surgical procedures seem to play a role in the anti-inflammatory effect. Goto at al. reported no effect on inflammation from volatile anesthetics for patients undergoing cataract surgery [34]. This study suggests that the inflammatory response for minor surgery is not significantly affected by volatile anesthetics.

The effects of volatile anesthetics on inflammation may have significant implications. Inflammation is a common pathological process involved in almost all diseases acquired in life. Inflammation is also involved in various processes during perioperative period, such as wound healing and infection prevention. Further studies are needed to determine the consequences of the volatile anesthetic effects on inflammation under various clinical conditions. In addition, current studies have mainly explored the role of NF-κB in these anesthetic effects. Further studies are needed to fully understand how volatile anesthetics can induce anti-inflammatory responses and neuroinflammation.

## Competing interests

The authors declared that they have no competing interests.

## Authors’ contributions

ZZ conceived the structure and content of the manuscript. FB drafted part of the manuscript. ZZ modified and completed the manuscript. Both authors read and approved the final manuscript.
